# Using relationship styles based on attachment theory to improve understanding of specialty choice in medicine

**DOI:** 10.1186/1472-6920-6-3

**Published:** 2006-01-11

**Authors:** Paul S Ciechanowski, Linda LM Worley, Joan E Russo, Wayne J Katon

**Affiliations:** 1 Department of Psychiatry and Behavioral Sciences, University of Washington, Seattle, Washington, USA; 2 Department of Psychiatry, University of Arkansas for Medical Sciences, Little Rock, Arkansas, USA

## Abstract

**Background:**

Patient-provider relationships in primary care are characterized by greater continuity and depth than in non-primary care specialties. We hypothesized that relationship styles of medical students based on attachment theory are associated with specialty choice factors and that such factors will mediate the association between relationship style and ultimately matching in a primary care specialty.

**Methods:**

We determined the relationship styles, demographic characteristics and resident specialty match of 106 fourth-year medical students. We assessed the associations between 1) relationship style and specialty choice factors; 2) specialty choice factors and specialty match, and 3) relationship style and specialty match. We also conducted mediation analyses to determine if factors examined in a specialty choice questionnaire mediate the association between relationship style and ultimately matching in a primary care specialty.

**Results:**

Prevalence of attachment styles was similar to that found in the general population and other medical school settings with 59% of students rating themselves as having a secure relationship style. Patient centeredness was directly associated, and career rewards inversely associated with matching in a primary care specialty. Students with a self-reliant relationship style were significantly more likely to match in a non-primary care specialty as compared to students with secure relationship style (OR = 5.3, 95% CI 1.8, 15.6). There was full mediation of the association between relationship style and specialty match by the specialty choice factor characterized by patient centeredness.

**Conclusion:**

Assessing relationship styles based on attachment theory may be a potentially useful way to improve understanding and counsel medical students about specialty choice.

## Background

Personality traits can significantly influence specialty choice in medicine [1,2] and may also play an important role in physicians' decisions to change their specialty [1]. Current Graduate Medical Education funding restrictions limit the number of years of residency training to those required within a single specialty, and may not provide full funding for training if residency specialty is changed mid-stream. As a result, medical schools work diligently to help prospective resident trainees make the best specialty choice by providing a variety of assessment and informational resources, including tools assessing personality traits [3-5]. A component of such resources often includes an assessment of one's comfort in participating in moderate- to long-term relationships in which important areas of care may include dealing with patients' intimate problems or interpersonal relationships [3,4]. Primary care specialties (e.g. family medicine, general internal medicine, pediatrics and obstetrics and gynecology [6]) often allow more opportunities for close relationships with patients due to continuity of care over many years.

Attachment theory plays an important role in personality development [7] and is rapidly becoming an important construct for understanding interpersonal aspects of medical care, the patient-provider relationship [8-11] and provider behavior [12-15]. The basic premise of attachment theory is that all individuals psychologically incorporate prior experiences with caregivers, forming enduring cognitive models of caregiving that persist throughout adulthood [16]. Relationship styles based on attachment theory are learned ways of interacting in interpersonal relationships throughout life and particularly at times of vulnerability (e.g. stress). All individuals are characterized by one of four predominant relationship styles derived from attachment theory: "secure", "cautious", "support-seeking" and "self-reliant" [17]. (These relationship styles were originally defined in the literature as secure, fearful, preoccupied and dismissing attachment styles, respectively [18]. We have used the current names for the latter three attachment styles as an effort to make the concept of attachment styles as accessible as possible to educators who are counseling students or residents, and for facilitating use by clinicians for the purposes of counseling with patients, or for self-reflection.)

Adults with a predominantly secure relationship style are generally comfortable depending on and being readily comforted by others. Adults with a predominantly self-reliant relationship style develop strategies at early stages in their development in which they become highly self-reliant [18] and are generally less comfortable trusting others. On the other hand, adults with a predominantly support-seeking relationship style are often more emotionally dependent on others' approval and support than those with the other relationship styles. Individuals with predominantly cautious relationship style share many of the characteristics of those with support-seeking relationship style in that they may desire to seek out social contact (i.e. they are not highly self-reliant), but this desire is simultaneously inhibited by fear of intimacy, often leading to approach-avoidance behavior [19].

We have previously shown that cautious and self-reliant relationship styles are less associated, and that secure relationship style, more highly associated with choosing primary care as a specialty by second year medical students [20]. In the current study of fourth year medical students, we set out to extend our findings by determining if relationship styles are associated with specific mediating factors that influence actual specialty match. We first planned to confirm that relationship-focused specialty choice factors are associated with specialty match – specifically, matching in primary care. Secondarily, we hypothesized that relationship styles would be associated with specialty choice factors, particularly by discriminating those which are relationship-focused from those which are not. Finally, we hypothesized that the association between relationship style and matching in a primary care specialty would be mediated by relationship-focused specialty choice factors.

## Methods

In April 2003, one hundred and six (of 129) fourth year medical students in attendance at a first meeting of their 'Last Chance Course', a month long course to prepare medical graduates for internship, at the University of Arkansas for Medical Sciences, College of Medicine, completed a questionnaire assessing relationship style based on attachment theory, factors potentially influencing specialty choice, and demographic data. Participating students consented to allowing researchers to have access to information about their recent residency specialty match from the College of Medicine. The University of Arkansas for Medical Studies institutional review board provided a priori approval for: the questionnaire; access, with student consent, to residency match records; and, all correspondence with students by the research team over the duration of the study.

### Self report instruments

1) Participants completed the Relationship Questionnaire (RQ) [21], which is an instrument measuring relationship style of respondents based on attachment theory. Four paragraphs are presented describing relationship styles and subjects are asked to choose the style that best suits them from secure, self-reliant, cautious and support-seeking relationship styles. The items have shown convergent and discriminant validity with other self-report measures and interview ratings [21]. Sample sentences from the paragraphs describing the four relationship styles include: Secure – "It is easy for me to get emotionally close to others", "I am comfortable depending on other people"; Self-reliant – "It is very important to me to feel self-sufficient", "I prefer not to have other people depend on me"; Cautious – "I am somewhat uncomfortable being close to others", "I worry that I will be hurt if I allow myself to become too close to others"; and Support-seeking – "I want to be completely emotionally intimate with others", "I find that others are reluctant to get as close as I would like".

2) Factors influencing specialty choice. Based on a literature review we derived the following 16 items characterizing students' reasons for choosing their specialty: specialty variety [22]; intellectual content or challenge [22]; research opportunities [22]; independence [3]; working with patients [3]; comfortable lifestyle [22-24]; financial rewards [3,22,24]; job opportunities [22]; societal need [22]; taking care of patients [3]; "keeping options open" [24]; prestige [3,24]; interaction with patients [23]; diversity of the patient population [23]; holism, continuity & prevention [23]; and establishing long term, in-depth relationships with patients [24]. Using a 5-point Likert scale ranging from "not at all" to "very strong influence" (see Table 2: Appendix) we asked students to rate the degree to which each of these was considered important as a reason for their specialty choice.

3) Demographic data were obtained and included age, gender, marital status, living situation and race.

4) Specialty choices were derived from resident specialty match list from the College of Medicine and were clustered into the following groups [3,23-25]: 1) Primary care: family practice, pediatrics, obstetrics and gynecology [6,26,27] and general internal medicine, and 2) Non-primary care: all non-primary care specialties (surgery and surgical subspecialties, internal medicine subspecialties, dermatology, geriatrics, psychiatry, anesthesiology, emergency medicine, pathology, diagnostic radiology).

### Statistical analysis

#### Baseline data

We analyzed data using SPSS 11.0 for Windows. We examined age and gender differences between survey respondents' and non-respondents' using t-tests for continuous data and chi-square tests with corrections for continuity for categorical data. We also used t-tests and chi-square tests to determine if there were differences in demographic characteristics between relationship style groups.

#### Specialty choice items

A principal component factor analysis using a varimax rotation with a Kaiser normalization was used to examine the factor structure and inter-relationships of the sixteen "specialty choice items". To determine the number of factors or dimensions of the specialty choice items, we chose principal components with eigenvalues greater than 1. We also chose items that met the criteria of simple structure, that is, they had a factor loading of 0.50 or greater on a single factor and very small factor loadings on the other factors. We eliminated two items (research opportunities and societal need), as they failed to load on a factor with any of the other items (see Table 1 and Table 2: Appendix). Based on the factor analyses, we created scales by summing up items loading on each factor. We examined the internal consistency reliability of the scales using Cronbach's alpha coefficient.

#### Mediation analyses

In order to determine if specialty choice factors mediate the association between relationship style and choice of primary care, we tested four conditions that must hold to show mediation [28]: 1) the independent variable [IV] (relationship style) must significantly affect the dependent variable [DV] (choosing primary care) when regressing the DV on the IV; 2) the IV (relationship style) must significantly affect the mediator (specialty choice factors) when the mediator is regressed on the IV; 3) the mediator (specialty choice factors) must significantly affect the DV (choosing primary care) when regressing the DV on both the IV and on the mediator; 4) the effect of the IV on the DV must be less when the mediator is controlled as in #3 than when it is not, as in #1.

For condition 1, we used logistic regression analysis with the outcome being a match in a non-primary care (1) versus primary care (0) specialty, and the predictor being the four categorized relationship style groups with the secure style group as the reference group. To examine condition 2, we used linear regression to determine if the relationship style groups (dummy variables for cautious, support-seeking and self-reliant relationship style with secure relationship style as the reference group) were related to each of the three specialty choice factors. To test condition 3 we fit a logistic regression model with both relationship style and the specialty choice factors that met condition 2, with the outcome being a match in a non-primary care (1) versus primary care (0) specialty. In this model, we examined the significance of the mediator and the change in the Wald's t to determine if mediation was demonstrated (condition 4). For the instances where mediation was demonstrated (when all four conditions were met), we calculated the proportion of the relationship between relationship style and match in a primary care specialty that was mediated by the specialty choice factors, using the methods of Shrout and Bolger [29]. This method allowed us to represent the strength of the mediation on a continuum of 0 to 100% rather than categorically as to whether mediation occurred or not.

## Results

There were no significant differences between respondents (N = 106) and non-respondents (N = 23) on age. However, significantly fewer females were represented among non-respondents (13%) than among respondents (40%) (Chi square = 6.0, p < .05).

### Relationship style groups

Overall, 59.4% of the student sample reported having a secure relationship style, with the remainder rating themselves as self-reliant (19.8%), cautious (10.4%) and support-seeking (10.4%).

### Demographic data

Mean age of the sample was 27.7 ± 3.9 years and the majority of the sample was male (60%) and Caucasian (91%). Fifty-seven percent of the sample said they were married or living as married and only 25% described themselves as living alone.

There were no significant differences in age, gender or race between relationship style groups. However, relationship style groups were associated with marital status and living situation. Patients with secure relationship style were more likely to report being married or living as married as compared to the other three styles (p < .01); there were significant post hoc differences between secure and cautious relationship style (70% vs. 36%, p < .05) and between secure and self-reliant style (70% vs. 33%, p < .004), but not between secure and support-seeking relationship style (70% vs. 46%, p = .12). Similarly, patients with secure relationship style were less likely to be living alone compared to the other three relationship styles (p = .03); there were significant post hoc differences between secure and cautious relationship style (16% vs. 46%, p = .04) and between secure and self-reliant style (16% vs. 43%, p = .01), but not between secure and support-seeking relationship style (16% vs. 18%, p = .60). We did not include these demographic variables in the remaining models as we did not feel that they were mediators of the studied associations.

### Specialty choice factors

The principal component factor analysis resulted in three factors with eigenvalues greater than 1.00 that accounted for 59.6% of the total item variance. Table 1 shows the results of the analysis. The first factor labeled "patient centered" describes specialty choice items most strongly characterized by the item "interaction with patients" and has 6 items with loadings > 0.55. The second factor labeled "career rewards" has 5 items with loadings > 0.54, and is most strongly characterized by the item on "financial rewards." The third factor labeled "intellectual aspects" contains three items with loadings > 0.53, and is best characterized by the item "specialty variety." The coefficient alphas for the scales ranged from excellent to moderate: patient centered factor = 0.90; career rewards factor = 0.69; and the intellectual aspects factor = 0.57.

### Relationship style and specialty choice factors

**Figure 1:**
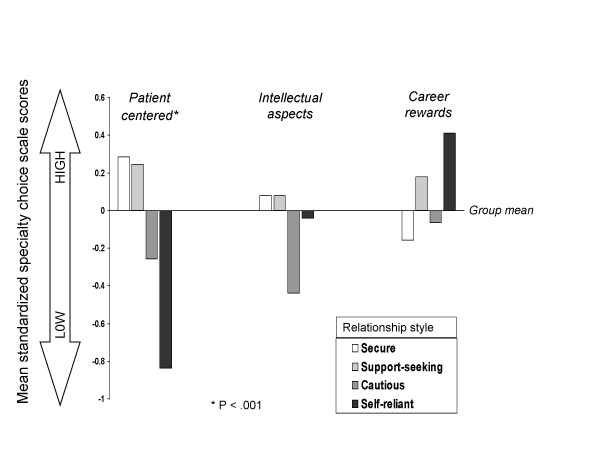
*The association of relationship styles and specialty choice scale scores*. Mean standardized specialty choice scale scores are illustrated for each relationship style in the specialty choice factor domains of patient centeredness, intellectual aspects and career rewards.

Figure 1 shows the profiles of the relationship styles by the three specialty choice scale scores. These results correspond with the linear regression analyses, which showed a significant difference between the relationship style groups on the patient centered factor [F(3, 101) = 8.6, p < .001], and no significant differences on the intellectual aspects [F(3, 101) = .86, p = .46] or career rewards [F(3, 101) = 1.8, p = .15] factors. As can be seen in figure 1, the significant differences between the relationship style groups on the patient centered factor was due primarily to the students with self-reliant relationship style having significantly lower patient centered factor scores than those with secure relationship style [t(101) = 4.9, p = < .001]. In comparison to patient centered factor scores in the secure relationship style group, the cautious relationship style group showed trend level lower scores [t(101) = 1.8, p = .07], while there was no significant difference in scores between support-seeking and secure relationship style.

### Relationship style and primary care specialty match

Logistic regression analyses revealed that the relationship style groups were significantly related to matching in a primary care specialty [Wald's test = 9.43, df = 3, p = .024], therefore condition 1 of mediation was established. Students with self-reliant relationship style were significantly more likely to match in a non-primary care specialty as compared to students with secure relationship style (OR = 5.3, 95% CI 1.8, 15.6). Support-seeking and cautious relationship styles were not significantly different from secure relationship style with regard to specialty match. Due to our finding that only the patient centered specialty choice factor scale was related to the relationship style groups, it was our only test of mediation. Because relationship style (the predictor) was not significantly related to the career rewards or intellectual aspect factors, they do not meet condition 2 for mediation. A second logistic regression showed that greater patient centeredness was significantly related to matching in a primary care specialty [Wald's test = 24.7, df = 1, p < .001], thus satisfying the third condition for mediation. [In separate bivariate models assessing specialty choice factors, greater endorsement of career rewards as a specialty choice factor was strongly associated with choosing a non-primary care specialty [Wald's test = 11.1, df = 1, p < .001], and intellectual aspects did not predict matching in either primary or non-primary specialty]. Lastly, in this model, relationship style was no longer statistically significantly related to matching in a primary care specialty [Wald's test = 1.76, df = 3, p = .63], after controlling for the patient centered specialty choice factor, because there was 100% mediation of the relationship between relationship style and matching in a primary care specialty by this factor. That is, students with self-reliant relationship style were no longer significantly more likely to match in a non-primary care specialty as compared to students with secure relationship style (OR = 1.1, 95% CI .26, 4.3).

## Discussion

The current study provides preliminary evidence for the utility of a specialty choice factor questionnaire derived from the literature [3,22-24]. Factor analysis of the specialty choice questionnaire identified three factors – patient centeredness, career rewards and intellectual aspects – that are associated with specialty choice. In bivariate models, patient centeredness is directly associated, and career rewards inversely associated with matching in a primary care specialty.

This study also demonstrates the potential utility of assessment of relationship styles in helping to determine how well prospective residents will suit a match in a primary care specialty. Our findings replicate some of our previous findings [20]. In this sample, relationship style prevalence, based on attachment theory, is similar to that in found in the general population [30] and in another medical school sample [20]. Also, in our previous study, second year students with a cautious style (OR = 5.9, 95% CI 1.9, 18.7) and students with a self-reliant style (OR = 2.4, 95% CI .96, 5.9) were more likely to say they would choose a non-primary over primary care specialty. In the current study we found that cautious style was not associated with specialty match. It is possible that the medical school samples were different in measured and unmeasured ways. Compared to our prior study sample, our current sample was more likely to be male (60% vs. 37%), Caucasian (91% vs. 79%) and married or living as married (57% vs. 31%). Also, the meaning of a specialty choice preference endorsed by second year students who have little or no clinical experience is unquestionably different compared to the specialty match decision of a fourth year student which is often painstakingly made after much consideration and which is inevitably influenced by intense clinical experiences in the senior years of medical school.

The fact that assessment of general relationships using a relationship style questionnaire was associated with a patient centered specialty choice factor essentially validates the utility of the relationship style measure in assessing medical providers. It may also suggest that personality factors, which are relatively fixed, may limit the effectiveness of many current initiatives to enhance training in patient centered care by changing provider's clinical approaches [31,32]. Instead, efforts may need to be placed in restructuring clinics so that providers who are innately patient centered may work together and augment the care of providers who are less inclined to provide patient centered care. Such efforts may ultimately lead to greater satisfaction among providers and patients.

Based on our mediation analyses the association between relationship style and match in a primary care specialty is fully mediated by the patient centered specialty choice factor. This supports the recognition that personality factors are associated with face-valid questions about medical practice preferences. Assessing relationship styles of prospective residents may serve as a useful tool in screening and recruiting program candidates who are well matched for satisfying careers in their chosen specialty. The use of relationship style questionnaires based on attachment theory may also characterize behavioral aspects of providers within the patient-provider relationship. Medical students sorted themselves into a range of responses regarding their perception of the importance of patient centered practices. Previous studies have shown that patients with self-reliant style are less likely to collaborate, to provide information [33], and are less likely to adhere to treatment [34]. The relationship style of providers may thus play an important role in the interactions that occur between patient and provider. Patients often see several primary care providers until they find a provider they are "comfortable" with; the matching of patient and provider relationship styles is unquestionably an important factor in this sense of "comfort".

There are several strengths of this study. As compared to our previous study, we assessed 4^th ^year medical students who were about to embark upon residency training. We also used definitive data about specialty choice based on a residency match list. Furthermore, we derived specialty choice questions from prior literature and found that these items combined into three common factors. Finally, we were able to replicate many of our previous findings in a medical school setting geographically removed from the previous sample. A limitation of this study is the relatively small sample size, which limits the degree to which we can examine the main specialties individually. Subsequent multi-site studies may help to increase sample size to allow individual specialties to be assessed in the way that groups of specialties based on matching in primary care were in the current study.

## Conclusions

In this study of 4^th ^year medical students, patient centeredness was directly associated, and career rewards inversely associated with matching in a primary care specialty. Students with a self-reliant relationship style were more likely to match in a non-primary care specialty as compared to students with secure relationship style and there was full mediation of this relationship between relationship style and specialty match by the specialty choice factor characterized by patient centeredness. Assessing relationship styles using attachment theory may be a potentially useful way to understand and counsel medical students about specialty choice.

## Competing interests

The author(s) declare that they have no competing interests.

## Authors' contributions

LW helped in design of the study, acquisition of data, analysis and interpretation of the data and helped with drafting of the manuscript. PC helped in design of the study, with analysis and interpretation of the data and in drafting the manuscript. JR helped in the design of the study, with analysis and interpretation of the data and in drafting the manuscript. WK helped the design of the study, with interpretation of the data and in drafting the manuscript.

## Pre-publication history

The pre-publication history for this paper can be accessed here:



## Figures and Tables

**Table 1:** Results of the Principal Component Factor Analysis. Rotated Component Matrix is depicted in which a principal component analysis was the extracting method and Varimax with Kaiser Normalization was the rotation method. Rotation converged in five rotations.

Factors	Items	Component		
		1	2	3

Patient centered	Interaction with patients	.92	-.02	.08
	Working with patients	.89	-.13	-.01
	Taking care of patients	.88	-.07	.04
	Establishing long-term, in-depth relationships with patients	.88	-.08	.06
	Holism, continuity and prevention	.74	-.10	.01
	Diversity of the patient population	.56	-.25	.27

Career rewards	Financial rewards	-.44	.70	.06
	Job opportunities	-.02	.67	.35
	Comfortable lifestyle	-.07	.65	-.13
	Independence	-.04	.62	.04
	Prestige	-.09	.55	.23

Intellectual aspects	Specialty variety	.19	-.11	.82
	Intellectual content or challenge	-.12	.19	.72
	Keeping options open	.18	.28	.54

**Table 2:** Appendix. Questionnaire assessing Factors Influencing Specialty Choice (FISC). People vary in the factors that influence their choice of specialty in medicine. Please rate each of the following factors as to the extent to which you think they factor into your choice of specialty:

	**Not at all**	**Somewhat**			**Very strong influence**
1. Specialty variety	**1**	**2**	**3**	**4**	**5**
2. Intellectual content or challenge	**1**	**2**	**3**	**4**	**5**
3. Independence	**1**	**2**	**3**	**4**	**5**
4. Working with patients	**1**	**2**	**3**	**4**	**5**
5. Comfortable lifestyle	**1**	**2**	**3**	**4**	**5**
6. Financial rewards	**1**	**2**	**3**	**4**	**5**
7. Job opportunities	**1**	**2**	**3**	**4**	**5**
8. Taking care of patients	**1**	**2**	**3**	**4**	**5**
9. Keeping options open	**1**	**2**	**3**	**4**	**5**
10. Prestige	**1**	**2**	**3**	**4**	**5**
11. Interaction with patients	**1**	**2**	**3**	**4**	**5**
12. Diversity of patient population	**1**	**2**	**3**	**4**	**5**
13. Holism, continuity & prevention	**1**	**2**	**3**	**4**	**5**
14. Establishing long term, in-depth relationships with patients	**1**	**2**	**3**	**4**	**5**

Coding for the Factors Influencing Specialty Choice (FISC): Patient centered: items 4,8,11,12,13,14; Career rewards: items 3,5,6,7,10; Intellectual aspects: items 1,2,9.
